# Effects of Immersive Natural Sound Stimulation With the Spherison Device on Anxiety Symptoms: A Case Series

**DOI:** 10.1002/ccr3.72214

**Published:** 2026-03-09

**Authors:** Donatella Marazziti, Gerardo Russomanno, Matteo Gambini, Riccardo Gurrieri

**Affiliations:** ^1^ Department of Clinical and Experimental Medicine, Section of Psychiatry University of Pisa Pisa Italy

**Keywords:** anxiety disorders, immersive auditory stimulation, natural sound therapy, nonpharmacological treatment, Spherison

## Abstract

Anxiety disorders are highly prevalent and often associated with incomplete response, poor tolerability, or refusal of pharmacological treatments. Nonpharmacological interventions targeting stress‐regulatory systems are therefore increasingly explored. We describe herein the clinical effects of immersive natural sound stimulation delivered via the Spherison device, based on Sound 6D technology, in patients with generalized anxiety disorder (GAD). We report a case series of three adults meeting DSM‐5‐TR criteria for GAD who refused or discontinued psychotropic medication. All participants underwent three weekly 30‐min sessions of passive listening to immersive natural soundscapes using the Spherison device. Anxiety severity was assessed at baseline and during treatment using the Beck Anxiety Inventory (BAI), Hamilton Anxiety Rating Scale (HAM‐A), and Clinical Global Impression (CGI). All patients showed a progressive and clinically meaningful reduction in anxiety symptoms across sessions. Improvements were observed on both self‐reported and clinician‐rated measures, with no adverse effects reported. Two patients remained clinically stable at 6‐month follow‐up. Immersive natural sound stimulation delivered through the Spherison device may represent a feasible and well‐tolerated nonpharmacological intervention for anxiety symptoms. Although limited by the small sample size and uncontrolled design, these findings support further controlled studies to evaluate efficacy and underlying mechanisms.

## Key Clinical Message

Immersive natural sound stimulation delivered via the Spherison device may represent a promising nonpharmacological intervention for anxiety disorders. This case series highlights its potential to induce a clinically meaningful reduction in anxiety symptoms, even in patients previously unresponsive or unwilling to use psychotropic medication.

## 1 Introduction

It is estimated that about one out of five adults suffer from anxiety symptoms, and approximately 4% of the global population meets the diagnostic criteria for an anxiety disorder, a category including generalized anxiety disorder (GAD), panic disorder, social anxiety disorder, agoraphobia, and specific phobias [1]. Psychotherapy and psychotropic drugs, specifically benzodiazepines and antidepressants, are currently the most widely used therapeutic strategies for anxiety disorders [2, 3]. Besides the current guidelines, a variety of more or less effective nonpharmacological approaches have been proposed with the aim of reducing anxiety symptoms through complementary mechanisms [4].

Music‐based and natural‐sounds interventions have increasingly been recognized as an effective nonpharmacological tool for the management of anxiety symptoms and disorders. A large body of research supports its benefits to modulate autonomic nervous system activity and promote psychophysiological relaxation across diverse populations, including healthy individuals and patients in clinical settings [5, 6, 7, 8, 9, 10, 11]. Indeed, structured auditory stimulation has been associated with a reduction in heart rate, blood pressure, respiratory rate, and skin conductance response, as well as a decrease in circulating cortisol levels [12, 13, 14, 15, 16, 17, 18, 19]. These physiological changes reflect a down‐regulation of sympathetic nervous system activity and an enhancement of parasympathetic tone, leading to a measurable state of relaxation [17, 20].

Subjective reports align with these biological findings. However, participants exposed to structured music interventions report significant decreases in anxiety scores, stress perception, and emotional tension compared to silence or nonmusical controls [15, 17, 21]. Notably, in perioperative settings, passive music listening demonstrated efficacy comparable to that of benzodiazepine premedication in reducing preoperative anxiety, while in critical care contexts, music exposure has been associated with reduced anxiety levels and lower sedative requirements [22, 23, 24, 25].

Moreover, recent meta‐analyses confirmed that passive music listening may yield moderate to large effects in reducing self‐reported anxiety across different clinical populations, including surgical patients, individuals undergoing cancer treatment, and psychiatric patients [8, 17, 21, 26]. These effects are thought to arise not only from the direct soothing properties of music, but also from its ability to engage cortical and subcortical circuits involved in emotional regulation, reward processing, and autonomic control [10, 14, 15, 16].

**FIGURE 1:**
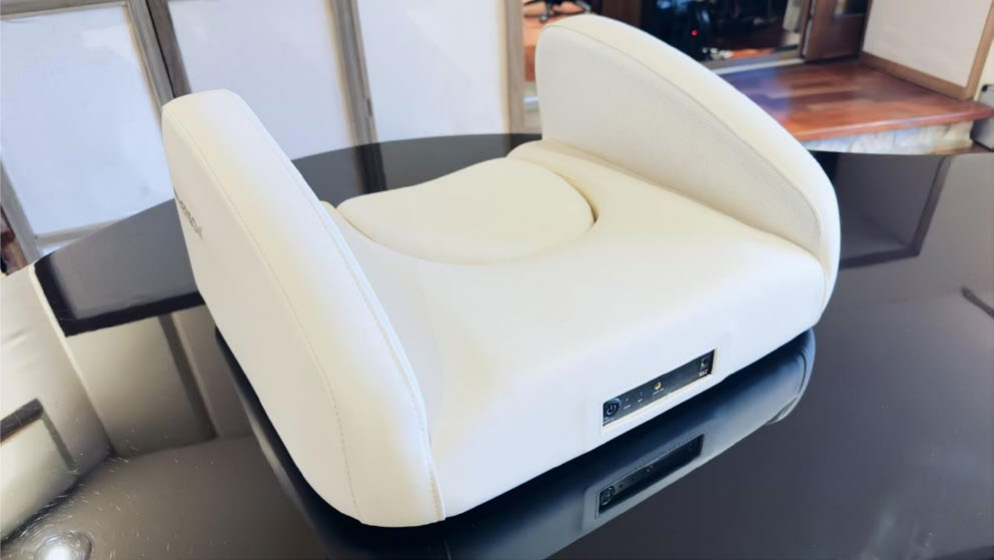
Spherison device.

In this context, the Spherison device, utilizing Sound 6D technology, offers a novel and advanced approach to immersive sound‐based therapy [27] (Figure 1).

Sound 6D technology refers to a multichannel auditory recording and reproduction system designed to capture and reproduce spatial, binaural, and vestibular‐related sound cues involved in natural auditory perception. Unlike conventional stereo or binaural recordings, this approach integrates multiple microphones positioned to record sound components associated with cochlear input, interaural differences, and vestibular‐related spatial orientation. It consists of two integrated components: Cocleo, a specialized recording microphone, and Spherison, a reproduction device designed to deliver six‐dimensional audio stimulation. Cocleo was developed based on a detailed anatomical and functional analysis of the human auditory system, extending beyond the cochlea to include the auricle, the surrounding bony structures, and the peripheral extensions of the acoustic and vestibular nerves. To replicate the natural mechanisms of sound localization, Cocleo integrates six microphone capsules (three for each side), capturing cochlear, binaural, and vestibular features of sound perception. The recording system is designed to capture multiple spatial and binaural components of natural soundscapes, with the aim of reproducing sound localization cues involved in natural auditory perception. Spherison, shaped like a pillow, is the device used to reproduce the Sound 6D audio contents. Two carefully positioned speakers deliver sound to all the anatomical structures involved in hearing, from the external ear to the Eustachian tubes, allowing the listener to perceive sound localization as it originally occurred during recording sessions.

A pilot study conducted at the University of Milano‐Bicocca demonstrated that Sound 6D stimulation provided through Spherison induced stronger physiological markers of relaxation, as measured by galvanic skin response (GSR) and photoplethysmography (PPG), compared to traditional headphone listening [28]. These findings support the potential role of immersive auditory technologies in promoting relaxation and reducing anxiety symptoms [28].

Building upon these preliminary findings, we present herein a case series exploring the effects of Sound 6D stimulation delivered via the Spherison device in individuals with clinically significant anxiety symptoms/disorders. All participants underwent three individual sessions, each spaced 1 week apart, of passive listening to natural environmental soundscapes (birds, whirling shakers, waves, cicadas), specifically recorded with the Cocleo microphone. Each session lasted 30 min, conducted in a dedicated, quiet, darkened room, where participants lay down in a supine position throughout the session and scheduled at fixed weekly intervals (every 7 days). A side panel was placed to shield participants from external visual distractions. The researcher's desk was positioned nearby but outside the participant's direct field of vision, allowing unobtrusive monitoring during the intervention.

To monitor anxiety severity and response to the intervention, three validated clinical instruments were used: the Beck Anxiety Inventory (BAI): a 21‐item self‐report questionnaire assessing symptom intensity over the past week. Total scores range from 0 to 63 and are classified as follows: 0–7 (minimal), 8–15 (mild), 16–25 (moderate), and 26–63 (severe anxiety); the Hamilton Anxiety Rating Scale (HAM‐A): a clinician‐administered scale with 14 items covering psychic and somatic anxiety. Scores range from 0 to 56, with < 17 indicating mild, 18–24 moderate, and ≥ 25 severe anxiety; the Clinical Global Impression (CGI): a clinician‐rated tool including two components: Severity (CGI‐S, 1 = normal to 7 = extremely ill) and Improvement (CGI‐I, 1 = very much improved to 7 = very much worse). Baseline assessments (T0) were performed immediately before the first session. Intermediate assessments (T1) were conducted 7 days later, prior to the second session, and final assessments (T2) were conducted 7 days after the second session, following completion of the third session. When available, follow‐up clinical evaluations were conducted approximately 6 months after completion of the intervention.

## 2 Case History

### 2.1 Case #1

Patient #1 was a 38 years‐old female teacher, married with two children, with a negative family history of psychopathological conditions. She has complained of living in a constant state of alertness since her late adolescence when she was attending her last year of high school. She consulted her physician, who prescribed benzodiazepines with benefits for about 6 months. However, at the beginning of university and the study load, the clinical picture worsened and was complicated by somatic symptoms (headache, gastric and muscle pain, agitation, fatigue, and initial insomnia). Therefore, the patient was referred to some psychiatrists who made the correct diagnosis of GAD and prescribed different drugs throughout the years, such as benzodiazepines (lorazepam, delorazepam, and diazepam) together with tricyclic antidepressants (imipramine and nortriptyline), and small doses of antipsychotics (quetiapine 25 mg/day). At the same time, she underwent unspecified psychotherapy with partial effect. She reported no current or past use of nicotine, alcohol, cannabis, or other psychoactive substances. When the patient consulted us, she had stopped all treatments some months ago, as she was convinced that all drugs were ineffective. After the explanation of the possible benefits of the Spherison device, she accepted to start this therapeutic strategy. The BAI and HAM‐A scores at baseline condition, before the treatment (T0), were, respectively, 31 and 28. The CGI score was 5, indicating marked illness.

### 2.2 Case #2

Patient #2 is a 30 years‐old, healthy single male chef with a university degree in nutrition. He recalled no family or personal history of neuropsychiatric disorders, except that his mother was “anxious” when she was an adolescent and went out with friends during the weekend and used to smoke small amounts of cannabis that was stopped 10 years ago. He denied current use of nicotine, alcohol misuse, or other psychoactive substances. The patient referred an overall wellbeing, effective coping strategies even when his father suddenly died, good social interactions and relationships, regular sport activity, and openness to life and projects for the future. When he finished university, after a series of internships, he had been working in different restaurants until a famous chef engaged him in his team, composed of about 30 individuals. It was a great challenge and, for the first time in his life, he started to be afraid that he could not accomplish the requested tasks, mainly because the job environment was very competitive. In spite of the great chef being satisfied and used to being commended for his creations, he could never relax and lived in a state of continuous worry and preoccupation about failing or making mistakes, tension, and restlessness. He consulted us when he noted difficulties in concentrating and sleeping. After informing him that his diagnosis was GAD, he said that he did not want to take any drug, as he was frightened of the possible onset of side effects, especially sedation. After detailed explanation of the Spherison immersive sound therapy protocol, the patient agreed to undergo this non‐pharmacological intervention. At baseline (T0), anxiety severity was classified as moderate, as the BAI score was 24, the HAM‐A score was 21, and the CGI‐S score was 4.

### 2.3 Case #3

Patient #3 is a 43 years‐old healthy divorced woman, with no children, working as a nurse in a geriatric hospital. Her family history is negative, but she referred to have been suffering from panic attacks after the birth of her second child 24 years ago, when she suffered from high levels of anxiety and panic attacks related to hyperthyroidism due to Hashimoto thyroiditis that was treated for some months and resolved. After some years, her husband left her and the children for another woman, but she could face the difficulties of the new family situation with the help of her parents, who took care of the nephews when she was on duty in the hospital. Some months ago, with no apparent reason, she experienced a decreased appetite, gastric pain, a constantly increasing sense of inner tension, difficult concentration at work and home, and trembling hands significantly interfering with daily activities. She underwent all necessary blood and urine tests that were all negative; in particular, she had no thyroid problem. She denied use of illicit substances and nicotine. She tried to self‐medicate with small doses of benzodiazepine (lorazepam 1 mg/day), which she used to take with a glass of wine in order to reach complete symptom control. After 2 months of this regimen and the need to increase the wine glasses to obtain the same effects, she decided to consult us. As she requested not to take drugs any longer, she was glad to be treated by means of the Spherison device. At baseline (T0), her anxiety severity was classified as severe: the BAI score was 29, the HAM‐A score was 26, and the CGI‐S score was 5.

## 3 Differential Diagnosis, Investigations and Treatment

All three patients met DSM‐5‐TR diagnostic criteria for GAD based on clinical interviews and symptom profiles, which included excessive and uncontrollable worry, restlessness, muscle tension, and impaired sleep or concentration lasting for more than 6 months. Other primary anxiety disorders (e.g., panic disorder, social anxiety disorder), mood disorders, and medical conditions (such as thyroid dysfunction) were ruled out through comprehensive clinical history and standard laboratory investigations. No patient was suffering from any comorbid psychiatric diagnoses or ongoing substance use disorders. In all cases, the clinical picture was consistent with GAD without psychotic features, and no current major depressive episode was detected.

Given the patients' refusal to use or continue psychotropic medications, either due to previous inefficacy (Cases #1 and #3) or fear of adverse effects (Case #2), a nonpharmacological approach was proposed. Each participant underwent a course of immersive natural sound stimulation using the Spherison device, which incorporates Sound 6D technology. The intervention consisted of three individual sessions, each lasting 30 min and spaced 1 week apart, with passive listening to natural environmental soundscapes, recorded with the Cocleo microphone and reproduced through the Spherison device. The sessions were conducted in a quiet, darkened room with participants lying supine and shielded from external stimuli to promote immersive sensory engagement. Throughout the intervention, no pharmacological agents or additional psychotherapeutic treatments were administered.

Anxiety symptom severity was systematically monitored after two sessions (T1) and after the third session (T2). The results are summarized in Table 1.

**TABLE 1:** Clinical assessment scores (BAI, HAM‐A, CGI‐S, and CGI‐I) at baseline (T0), after two sessions (T1), and after three sessions (T2) for each patient undergoing Sound 6D stimulation via the Spherison device.

Patient	BAI_T0	BAI_T1	BAI_T2	HAM‐A_T0	HAM‐A_T1	HAM‐A_T2	CGI‐S_T0	CGI‐I_T1	CGI‐I_T2
P1	31	20	11	28	19	12	5	3	2
P2	24	14	6	21	13	7	4	2	1
P3	29	21	13	26	17	10	5	3	2

Abbreviations: BAI = Beck Anxiety Inventory (range: 0–63); CGI‐S = Clinical Global Impression—Severity; CGI‐I = Clinical Global Impression—Improvement; HAM‐A = Hamilton Anxiety Rating Scale (range: 0–56).

## 4 Conclusion and Results (Outcome and Follow‐Up)

All three patients experienced a progressive and clinically meaningful reduction in anxiety symptoms throughout the three‐session immersive natural sound intervention delivered via the Spherison device.

Patient #1 (female, 38 years): Initially classified as severely anxious, showed consistent improvement across all measures. BAI scores declined from 31 (T0) to 20 (T1) and 11 (T2), while HAM‐A scores decreased from 28 to 19 and then 12. CGI‐I improved from 3 (minimally improved) at T1 to 2 (much improved) at T2. The patient reported a subjective sense of relaxation and significantly improved sleep quality, particularly after the second session.

Patient #2 (male, 30 years): Presented with moderate anxiety at baseline. Following the first session, he reported immediate symptom relief. BAI scores dropped from 24 (T0) to 14 (T1) and then 6 (T2), while HAM‐A scores decreased from 21 to 13 and 7. CGI‐I scores improved from 2 (much improved) to 1 (very much improved), indicating a near‐complete resolution of symptoms.

Patient #3 (female, 43 years): Initially exhibited severe anxiety, with BAI and HAM‐A scores of 29 and 26 at baseline. These improved to 21 and 17 at T1, and to 13 and 10 at T2, respectively. CGI‐I ratings improved from 3 to 2. The patient noted that while the first session produced temporary relief, the second and third sessions resulted in sustained improvements and enhanced daily functioning.

Notably, no adverse effects were reported during or following the intervention. Two patients (Cases #1 and #2) were followed up for 6 months postintervention and remained clinically stable without recurrence of anxiety symptoms. During follow‐up clinical interviews, patients were asked about major changes in life circumstances, including interpersonal relationships, occupational conditions, and lifestyle factors. No relevant changes were reported during the follow‐up period.

These preliminary outcomes suggest that immersive sound therapy using the Spherison device may offer a safe and effective nonpharmacological alternative for individuals with GAD who are unresponsive to or refuse conventional treatments.

## 5 Discussion

Anxiety symptoms and/or disorders are a major problem worldwide, significantly impairing individual life and adjustment, while even representing a burden to social health systems. Although there exist different psychotherapeutic and psychopharmacologic approaches to manage these conditions, there is a proportion of nonresponders or those who misuse or abuse benzodiazepines, with their well‐known side effects. For these reasons, there is an increasing need and request for novel strategies, possibly grounded on a neurobiological background or hypothesis. Recently, much attention has been increasingly devoted to explore the benefits of music and natural sounds that seem to decrease the stress system and related disorders, including anxiety and depression, as well as to improve cognition, health, and the overall sense of wellbeing. Again, natural sounds seem to provoke a faster improvement than music. Although real data in clinical populations are limited, nevertheless some data show a decreased function of the brain areas regulating the fight/flight response [29, 30, 31] of the sympathetic nervous system and activation of the parasympathetic pathway and reward system [32, 33, 34].

The present paper describes the positive effects of Spherison, a tool of immersive sound‐based therapy [27]. As compared with similar instruments, Spherison possesses two carefully positioned speakers delivering sound to all the anatomical structures involved in hearing, from the external ear to the Eustachian tubes, allowing the listener to perceive sound localization. The patients recruited were two women and one man suffering from GAD who refused to take classical psychotropic drugs that they had tried in the past and did not want any longer for being ineffective (Patient #1 and #3), or for the fear of the sedative effects (Patient #2). All the patients reported a significant improvement since the first session, that became more robust at the end of the third session, as shown by the rating scales total score. They referred a sharp decrease in both physical and mental symptoms, an overall sense of relief, improving daily attention, with no side effects.

Two patients continued to be followed for the next 6 months and continued to be healthy with no previous anxious symptoms.

Although these results are intriguing, we highlight the main limitation of this report, specifically, the possible placebo effect of the treatment given the high expectation of the patients and/or positive expectancy toward the intervention. In any case, our opinion is that the possible use of Spherison (and other similar tools) in clinical settings should be promoted. Therefore, further studies should be carried out in larger samples and, at the same time, the neurobiological bases of music/natural sound therapies should also be deepened.

## Author Contributions

**Donatella Marazziti:** conceptualization, data curation, formal analysis, funding acquisition, investigation, methodology, project administration, resources, software, supervision, validation, visualization, writing – original draft, writing – review and editing. **Gerardo Russomanno:** data curation, formal analysis, methodology, software, supervision, writing – original draft. **Matteo Gambini:** data curation, formal analysis, methodology, visualization, writing – original draft. **Riccardo Gurrieri:** conceptualization, formal analysis, methodology, writing – original draft, writing – review and editing.

## Funding

The authors have nothing to report.

## Ethics Statement

All identifying information has been removed to ensure anonymity. The study was conducted in accordance with the Declaration of Helsinki.

## Consent

In accordance with the journal's policy on patient consent, written informed consent was obtained from all three patients included in this case series.

## Conflicts of Interest

The authors declare no conflicts of interest.

## Data Availability

The data that support the findings of this study are available from the corresponding author upon reasonable request.
